# The WHO Pandemic Agreement’s Missing Epistemic Architectures: Infodemics and Antimicrobial Resistance as Examples

**DOI:** 10.1007/s41649-025-00387-9

**Published:** 2025-07-16

**Authors:** Calvin Wai Loon Ho, Karel Caals

**Affiliations:** 1 School of Law, Monash University, Melbourne, Australia; 2 PHG Foundation, University of Cambridge, Cambridge, UK; 3 Centre for Medical Ethics and Law, Faculty of Law & Li Ka Shing Faculty of Medicine, University of Hong Kong, Hong Kong; 4 Monash Bioethics Centre, School of Philosophical, Historical and International Studies, Faculty of Arts, Monash University, Melbourne, Australia

**Keywords:** Global health, Epistemic justice, Infodemiology, Antimicrobial resistance, Pandemic Agreement, Equity, Infodemic

## Abstract

On 20 May 2025, the 78^th^ World Health Assembly adopted the World Health Organization’s Pandemic Agreement (PA). With the benefit of lessons learnt from the COVID-19 pandemic, the PA rightly focuses on advancing equity, but we are concerned that the PA appears to apply equity narrowly as distributive justice and neglects epistemic justice. Using infodemics and antimicrobial resistance (AMR) as examples, we argue that the PA misses epistemic architectures. We first explain why infodemics are an important public health concern that the PA seeks to address, even though it does not clearly mention them. We then explain why equity must be interpreted to include epistemic justice. Using infodemics as an example, we subsequently discuss how the epistemic architecture of the PA on infodemics will need to be set out clearly as an annex to the PA or through the adoption of an additional protocol. We note in particular that the PA could help to draw together different normative and human rights approaches and frameworks to meet the requirements of epistemic justice. A similar challenge applies to AMR as an epistemically complex phenomenon, and our argument is that a global response to AMR will require a just and equitable epistemic architecture that the PA could lay the foundation for.

## Introduction

On the 19^th^ of May 2025, the main provisions of the World Health Organization’s (WHO) Pandemic Agreement (PA) (WHO 2025a) were adopted by Member State delegations. The following day, the PA was adopted in a plenary session of the 78th World Health Assembly, paving the way for the creation of the third international law instrument that is specific to health, after the International Health Regulations (IHR) (WHO 2005) and the WHO Framework Convention on Tobacco Control (WHO 2003). This achievement comes a little over 2 years after the WHO ended the declaration of public health emergency of international concern (PHEIC) for COVID-19 on 5 May 2023. The degree of consensus is impressive, with 124 votes in favour, 11 abstentions and no objections.

There are a number of important accomplishments in the PA. A key feature is a mechanism that seeks to ensure equitable access to vaccines, diagnostics and therapeutics during a pandemic. Specific details of this mechanism, referred to as the Pathogen Access and Benefit Sharing system (PABS), are still being negotiated, but the plan moving forward is to set them out as an annex to the PA. Once the World Health Assembly adopts the PABS annex (scheduled for the next World Health Assembly in 2026), the PA will then be open for signature and consideration of ratification, including by national legislative bodies. After 60 ratifications, the PA will enter into force. Another important feature of the PA is the adoption of the One Health approach, which recognises that “the health of humans is closely linked and interdependent with the health of domestic and wild animals, as well as plants and the wider environment (including ecosystems), aiming for a sustainable balance, and uses an integrated multisectoral and transdisciplinary approach to pandemic prevention preparedness and response, which contributes to sustainable development in an equitable manner” (Article 1(b)). In this vein, the provisions of the PA on pandemic prevention and surveillance are more holistic and multifaceted. Implicit to these provisions are concerns around what has become known as infodemics (explained below) and antimicrobial resistance (AMR), which occurs when pathogens no longer respond to antimicrobials like antibiotics and antifungal medication. Both concerns are mentioned in the preamble of the PA, which highlights the importance of preventing misinformation, disinformation and stigmatisation through timely sharing of information, and the public health impact of the spread of AMR. Apart from these, the PA makes no further mention of misinformation, disinformation or stigmatisation, although there are some provisions relating to antimicrobials (but not AMR).

With the benefit of lessons learnt from the COVID-19 pandemic, the PA rightly focuses on advancing equity and other principles like solidarity, which have not found expression in the other two international health law instruments. However, we are concerned that the PA appears to apply equity narrowly as distributive justice, when the requirements of epistemic justice should also be observed. In the section that follows, we first explain why infodemics are an important public health concern that the PA seeks to address, even though there is no mention of this in any of its provisions. We then explain why equity must be interpreted to include epistemic justice, and using the example of infodemics, we subsequently discuss how the epistemic architecture (discussed below) of the PA on infodemics will need to be set out clearly,^1^ either in a manner that is similar to the PABS (i.e. by setting out explicit terms in an annex to the PA) or through the adoption of an additional protocol. We note in particular that the PA could help to draw together different normative and human rights approaches and frameworks to meet the requirements of epistemic justice. A similar challenge applies to AMR as an epistemically complex phenomenon, and our argument is that a global response to AMR will require a just and equitable epistemic architecture that the PA could lay the foundation for.

## Equity as a Key Guiding Principle

In this section, we explain why the PA is correct to focus on equity, especially during a public health emergency. However, its provisions are most clearly concerned with distributive justice, and less explicitly, digital justice as well, where they relate to the sharing of information or data. Critics point out that the PA does not go far enough to advance global justice in its distributive sense, but we highlight why the failure to address epistemic justice explicitly should also be a concern, particularly in relation to the challenges of infodemics. The concept of epistemic justice in the context of an infodemic is explained in the next section.

As noted above, the PABS system that is broadly articulated in Article 12 of the PA is a key enactment intended to secure distributive justice as equity during a pandemic emergency (Privor-Dumm et al. 2023). This system formalises the pandemic influenza preparedness framework, which currently lacks legal standing (Lajaunie and Ho 2018; Ho 2022). If adopted, the PABS system seeks to ensure that participating manufacturers make available to the WHO a target of 20% of their real-time production of vaccines, therapeutics and diagnostics for the pathogen causing the pandemic emergency. A minimum threshold of 10% of their real-time production is to be made available to WHO as a donation, and the remaining percentage, based on the nature and capacity of each participating manufacturer, is reserved at affordable prices to WHO (PA, Article 12(6)). The distribution of these products will depend on public health risk and need, paying particular attention to developing countries and the Global Supply Chain and Logistic Network (set out in Article 13 of the PA). Benefit-sharing provisions are to be included in the PABS system, which Article 12(8) identifies as capacity-building and technical assistance; research and development cooperation; facilitating rapid access to available vaccines, therapeutics and diagnostics with a view to responding to public health risks and events; granting of non-exclusive licences to manufacturers in developing countries and other forms of transfer of technology as mutually agreed, including transfer of relevant knowledge, skills and technical expertise. In the preamble, capacities and resources include technological, technical and digital health resources (WHO 2025a, 7). Other conditions or “elements” that ensure the fair, transparent, accountable, equitable and effective operation of the PABS system should continue to apply (Article 12(9)).

The centrality of equity as a normative goal of the PA should come as no surprise. Deep structural injustices and health inequalities across well-known indicators like income, occupation, gender, age and ethnicity hindered timely and equitable access to health products during the COVID-19 pandemic with the outbreak and cross-border transmission of SARS-CoV-2 from around the beginning of 2020 (see for instance, Ramraj 2021). The preamble of the PA expresses “deep concern” over the inequities at national and international levels and seeks to address these “serious shortcomings” at the national, regional and global levels in prevention, preparedness, response and health system recovery for public health emergencies of international concern (PHEIC), including pandemic emergencies. Although the implementation of the PA is to be guided by principles that include the sovereign right of States; full respect for the dignity, human rights and fundamental freedoms of all persons (including the enjoyment of the highest attainable standard of health, right to development and full respect from non-discrimination, gender equality and protection of persons in vulnerable situations); full respect for international humanitarian law; solidarity with all people and countries in the context of health emergencies and best available science and evidence, equity is clearly intended as the cornerstone principle (PA, Article 3). While the adoption of the PA is a much needed boost to global health multilateralism since the withdrawal of the United States (WHO’s largest donor) from the WHO (Cheng and Weissert 2025; Nature 2025), the PABS system has been criticised for not going far enough “to ensure that high-income countries and private companies behave fairly, that they do not stockpile millions of excess doses of vaccine or refuse to share life-saving knowledge and products, and that there are mechanisms to ensure that countries work together rather than against each other” (Lancet 2024). More generally, the PA lacks clear and concrete enforcement mechanism, funding obligations or provisions on inclusive, accountable and transparent public governance—all of which were impediments to effective and equitable response to the COVID-19 pandemic (Strobeyko et al. 2024; Moon et al. 2022). Compared with the working draft of the PA, one textual analysis finds that obligations tied to equity and human rights were less specific, which reflected a shift from cosmopolitan intentions and a reduced emphasis on cooperation (Anderson et al. 2025). Consequently, international observers like Human Rights Watch (2025) have depicted the PA as “a landmark, but flawed”.

In our view, one such flaw is the lack of engagement with epistemic justice, even though many provisions relate (albeit indirectly) to digital justice, i.e. fair and equitable access to, as well as the sharing and/or use of, digital technologies and information (see, for instance, PA Articles 6(3), 8(3), 9, 10(2), 11(1) and (5), 12, 14(7), 16, and 18(4)(c)). What is arguably different about the COVID-19 pandemic is the rapid dissemination of information through the pervasive use of the Internet and social networking sites like Facebook, Twitter/X and WhatsApp groups (Ho et al. 2020). These digital platforms and tools make available vast amounts of often conflicting evidence and opinions on the nature of SARS-CoV-2, public health countermeasures (including vaccines and other precautionary and treatment options), healthcare providers, as well as people and communities of particular ethnicity, among others. The failure to ensure fairness in how knowledge is produced, disseminated and valued during the COVID-19 pandemic has in turn led to stigma of individuals and communities, erosion of trust in professional organisations and health authorities, affected mental health and negatively influenced health decisions and behaviour. We highlight a few studies as illustration. In their analysis of news articles from four major Chinese media outlets from January 2020 to March 2023, Wang and Zhang (2025) show that conflicting media narratives could have contributed to stigma suffered by COVID-19 survivors. In the Australian cities of Sydney and Melbourne, media analysis from January 2020 to September 2021 by Nichole Georgeou et al. (2023) indicates that lockdown in lower socioeconomic areas with high migrant populations was stricter than more affluent areas. This could be due to media reporting in four major newspapers that portrayed people from lower socioeconomic areas as having a heightened risk of exposure to COVID-19. A similar phenomenon was reported in Hong Kong, where mental health and wellbeing of already marginalised or disadvantaged populations were disproportionately impacted by strict lockdowns (Cheung and Ip 2020). Misinformation that was disseminated through the internet, social media and other communication outlets was found to have perpetuated beliefs that led to vaccine avoidance, mask refusal and utilisation of medications with insignificant scientific data in a literature review conducted by Maria Mercedes Ferreira Caceres et al. (2022). Ballalai and Lins (2025) illustrate how vaccine misinformation disseminated by political figures in Brazil negatively impacted healthcare professionals and health decisions. In the USA, trust in physicians and hospitals was reported to have declined from 71.5% in 2020 to 40.1% in 2024 due to factors like health information overload, increased social and political divide and poor health literacy (Tandar et al. 2024; see also Perlis et al. 2024).

In the light of these challenges, the PA is right to recognise “the importance of building trust and ensuring the timely sharing of information to prevent misinformation, disinformation and stigmatization” (WHO 2025a, 8). Unlike the earlier drafts of the PA, however, there is no mention of, or engagement with, the inequities or injustices that are associated with this phenomenon, which more recently has been referred to as an infodemic. For instance, a draft instrument that was considered by the WHO’s Intergovernmental Negotiating Body in 2023 highlighted the need to “combat the infodemic, and tackle false, misleading, misinformation or disinformation” through measures that include (1) promoting and facilitating the development and implementation of risk communication, community engagement, infodemic management and educational and public awareness programmes; (2) conducting regular community outreach, social listening and periodic analysis and consultations with civil society organisations and media outlets; (3) promoting communications on technical advances that support the development and implementation of national and international rules and guidelines for pandemic prevention, preparedness, response and recovery of health systems and (4) taking effective measures to increase digital health literacy among the public and within the health sector (WHO 2023, Article 18). In the PA, however, there is no longer a specific provision on infodemics. In the next section, we first explain what an infodemic is, and then consider what epistemic justice is concerned with during such a phenomenon.

## Epistemic Justice during an Infodemic

The WHO defines an infodemic as “overabundance of information, accurate or not, in the digital and physical space, accompanying an acute health event such as an outbreak or epidemic” (WHO and UNICEF 2023, iv).^2^ Over the course of the COVID-19 pandemic, an accompanying infodemic gained visibility through the widespread use of digital resources, platforms and tools to support a range of communication, social interactions, activities and pandemic countermeasures. The WHO has been pivotal in framing and operationalising COVID-19 infodemic countermeasures through initiatives that monitor harmful information, and counter false information. It launched “infodemic management”, comprising of practices underpinned by the science of infodemiology—a relatively new specialty in epidemiology—and practices like social listening that seek to “listen” to community concerns and questions, promote understanding of risk and authoritative health information, build information resilience and empower (through engagement with) communities to take positive action. In 2023, the WHO published a technical manual on how an infodemic insight process could be initiated by public health agencies to learn about narratives circulating in the community (WHO 2023). These narratives and their level of risks are then described in an infodemic insight report, along with recommended actions that may be taken to protect public health. Since an infodemic insight report is generated rapidly through an integrated analysis of diverse data sources, it is not meant to replace more comprehensive evidence generation, such as in routine health programming. From a systems perspective, this infodemic insight process may be seen as falling within the broad scope of the IHR and related normative documents (WHO 2017) which mandate WHO Member States to detect and report potential PHEIC to the WHO. Where the PA is concerned, a High Contracting Party (i.e. a WHO member state that is a party to the PA) is required to progressively develop or strengthen measures and capabilities at the community level to prevent, detect and report unusual public health events to relevant authorities within its territory in order to facilitate actions for early containment at the source (Article 4(d)). At the time of writing, it is unclear if amendments to the IHR will render infodemics an explicit concern (PA, Article 1(c) footnote 2), or if an annex or additional protocol will be subsequently added to the PA to address this concern. The view that we put forward here is that infodemic is a concern that the PA seeks to address, even though there is no explicit mention of this term. If correct, we first explain the need to interpret the principle of equity as encompassing epistemic justice. We then address, in the sections that follow, at least two challenges that arise in relation to the One Health approach that the PA seeks to apply, notably in response to AMR.

Broadly speaking, epistemic justice has at least three concerns (Anderson 2012; Habgood-Coote 2025). First, it seeks to prevent or redress wrongs to a person as a knower that arise when the credibility of their judgments is undermined by negative identity or prejudicial stereotypes. In this sense, it seeks to prevent epistemic oppression that arises from unfair bias or discrimination (Fricker’s testimonial injustice) and from the inability to express social experiences due to the lack of conceptual resources (Fricker’s hermeneutic injustice) (Fricker 2007). The response to epistemic oppression is now understood broadly as including the aim of decolonising knowledge practices in order to foster more equitable knowledge systems (Cummings et al. 2025). Second, it seeks to ensure equality in the distribution of epistemic goods like truth, knowledge, understanding, justification and coherence. These goods are qualities or states of knowledge that are valuable in themselves and should be pursued for their own sake. Third, it is concerned with inverted epistemology, where an epistemic system cultivates ignorance that undermines the epistemic agency of the individuals concerned, typically to serve the interests of exploitative or oppressive systems. As Charles Wade Mills (1997, 18–19) explains (in critical race theory), white racial ignorance (or broadly speaking, the disregard by white persons of the challenges that non-white persons may face) is an epistemology of ignorance that maintains racist structures as it prevents white persons from understanding the world they themselves have made. For instance, the epistemology of ignorance depicts the epistemic basis of the “White supremacy” belief, which justifies and replicates mistaken perceptions of racial superiority or historical progress, by obstructing genuine understanding of European colonialism and how racial classifications have been used to justify social hierarchies and exploitation in Western modernity. Ironically, the workings of such an epistemology of ignorance are clearest to those (non-White persons) whose experience and interests contradict the misrepresentations of “White supremist” beliefs. In her discussion of Fricker’s and Mills’ concerns, Kimberly Hutchings (2023) explains that both associate epistemic injustice with the systematic marginalisation and exclusion of certain categories of people from the prevailing knowledge economy. While Fricker and Mills seek to produce better understandings of the world, their analytical approach differs in that the former seeks to negate bias and to increase a common pool of resources for knowledge, the latter focuses on positionality (as a privileged or marginalised knower) to knowledge production.

During the COVID-19 pandemic, we have illustrated how epistemic ignorance could have prevented better-off members of a society to understand the impact of harsh pandemic countermeasures on the vulnerable and marginalised. Cristian Timmermann (2020) explains that epistemic injustice is apparent in the lack of sensitivity towards the needs and circumstances of poorer members of society in public health policies and interventions. Epistemic injustices were also apparent in the new and invasive forms of information gathering and surveillance applied to support a wide range of pandemic countermeasures. In the Australian state of New South Wales, Katrina Hutchison and Jane Johnson (2023) provide one such account that arose when the word of some individuals was treated as highly credible whereas the word of members of marginalised groups was not. This in turn affected the geographical locations where surveillance measures were implemented, how these measures were enforced and the way that surveillance data was interpreted, especially when surveillance data was inconsistent with the individual’s own account. During the 2021 COVID-19 Delta outbreak, contact-tracing in NSW relied on a combination of personal interviews with individuals who contracted the disease, with information from different surveillance sources. These individuals suffered testimonial injustice when they were unjustly deemed to not know how they contracted the disease or suffered other related health conditions when they did, because of prejudicial classist, sexist or racist identity stereotypes (Hutchison and Johnson 2023, 85). A related notion is that of testimonial smothering (Dotson 2011), which occurs when an individual from an oppressed group modifies her testimony because she does not think her account would be taken seriously or believed. Conversely, public officials might have harboured prejudicial expectation that individuals (typically those with marginalised social identities) would give flawed testimony because the officials knew that they were not trusted.

With the digitalisation of public health interventions, notably in the use of contact-tracing software applications during the COVID-19 pandemic, technological systems easily become complicit in epistemic oppression. Technological redlining, where technology reinforces oppressive relationships and enacts forms of racial or gender-based profiling, and the proliferation of “fake news” that racialises ignorance about marginalised groups, are some well-known examples of epistemic harms that could arise from the inappropriate and unchecked use of digital technology. Furthermore, online platforms also perpetuate inverted epistemology through self-radicalisation (Noble 2018) and spread misinformation, particularly during public health crises (Duzen et al. 2023). Unless clear and effective safeguards are in place, the increasing reliance on Big Data and artificial intelligence (AI) capabilities for public health surveillance could present considerable epistemic harms to minority and marginalised groups and undermine equity and trust. Although the PA calls for High Contracting Parties to strengthen measures and capabilities progressively for pandemic prevention and coordinated multisectoral surveillance, it requires all parties to develop and adopt guidelines, recommendations and other non-binding measures as necessary, and to ensure consistency with the IHR (Article 4(4)). This concern is emphasised in an ethics guidance document published by the WHO on the use of social listening in infodemic management (WHO 2025b). Epistemic justice is identified as a substantive principle that should be reflected in social listening and other infodemic management practices, alongside procedural principles that seek to anticipate potential conflicts between epistemic justice and other epistemic values. However, it is less clear how the concerns of epistemic justice could be addressed in national laws and policies without the benefit of more detailed guidance within the PA framework.

## Epistemic Architecture

Let us briefly take stock of our discussion so far. The PA seeks to enhance global preparedness and response to future pandemics by building resilience, supporting prevention and detection capabilities, ensuring equitable access to countermeasures and improving global coordination. Equity is set out as the key guiding principle, but as we have explained, the provisions of the PA are most directly concerned with distributive justice, although digital justice is implicitly addressed as well. Our concern is the PA’s silence on epistemic justice even though its preamble highlights infodemics as a challenge. In the sections above, we have explained what the requirements of epistemic justice are and why they need attention. Epistemic injustices have been experienced by many during the COVID-19 pandemic, and many more are likely to be affected in future pandemics, with the prospect of continuing advancements in information and communications technology and increasing digitalisation, which the PA itself encourages. In this section, we first set out three reasons as to why the PA should also aim to advance epistemic justice, and to provide guidance on the measures that High Contracting Parties should consider and adopt in their laws and policies. We then explain how this could be done, which may be by linking up different normative frameworks that are already in place within a wider epistemic architecture (further discussed below). We proceed to set out our reasons.

First, epistemic evaluation of a public health system—along with an increasingly pronounced digital overlay in many systems—is complex. Evaluating a public health system from an epistemic point of view would involve at least considerations of individual epistemic outcomes, institutional properties and social justice (Habgood-Coote 2025). There is no agreement as to how the distortional effects of political power and domination (including political values that digital technology embodies as sociotechnical assemblage) on the proper operation of public health as an epistemic system should be accounted for. In an ideal epistemic public health environment, epistemic agents interact freely and equally and engage in public reasoning to discuss and deliberate common issues or challenges. In non-ideal settings however, certain individuals or groups that are already excluded are likely to suffer further oppression in such an environment. Writing in the context of women and minority groups, Nancy Fraser (1990) argues that a different discursive space, such as a network of counter-public spaces, may be more effective in ensuring epistemic justice. The perceived need to address upfront epistemic challenges of real-life systems has led to calls for non-ideal epistemology, as a means of moving away from assumptions like idealisation about agents and interactions among them, about social institutions and about the epistemic environment. For instance, Robin McKenna (2023) argues for epistemic explanation and evaluation to properly account for agents’ identities and the contexts they find themselves in. In his view, intellectual resource is misallocated if the focus is only on idealised conditions when there are pressing real world challenges to be addressed, since an idealised model may be ill-suited to guide action and that prescriptions from idealised models in the real world may be useless.

Second, trade-offs between different epistemic goods are often required; hence, guidance should be provided as to how different epistemic desiderata of a public health system are to be selected. The inability to articulate these desiderata limits epistemic accountability, given the lack of clarity as to when non-epistemic costs should be imposed on epistemic vice or bad epistemic practices like sharing false information. While accountability measures like call-outs of unreliable users or of users that fall short of epistemic norms are already implemented in certain digital platforms or environments, the problem of unequal distribution of costs for flouting shared epistemic norms remains unaddressed. Additionally, the problem of self-exclusion or self-silencing—particularly by the already disenfranchised and marginalised in society—persists. Existing epistemic injustices continue to undermine users’ epistemic agency by making discursive spaces inhospitable environments. In such environments, the specialised knowledge of marginalised people continues to be disregarded, which in turn lowers the diversity of epistemic communities. On this challenge, the PA remains conservative in its expressed preference for “the best available science and evidence as the basis for public health decisions for pandemic prevention, preparedness and response” (Article 3(6)). The failure to account for individual psychological factors and societal phenomena like hyperpolarisation, structural shifts in the media and public mistrust of elites may be an indication of epistemic injustice that could undermine the aims and effectiveness of the PA (Kirk 2024). To advance epistemic justice, there is arguably a greater need to focus on marginalised groups in systems-oriented social epistemology. As we shall see, this is also a human rights requirement. Otherwise, adding human oversight as a means of advancing epistemic accountability could result in human-driven inverted epistemologies.

Third, greater clarity is needed as to how international human rights law and mechanisms could be drawn on to at least mitigate or avoid epistemic injustices in pandemic prevention, preparedness and response. This failing was already evident during the COVID-19 pandemic when other information disorders that drove political, economic or other motivations were not adequately accounted for and addressed in infodemic management. Where the agencies of the United Nations (UN) are concerned, this problem is in part a flaw in the global architecture. The UN system’s response to the COVID-19 infodemic involved different UN agencies, including the WHO, the UN Department of Global Communications, UNESCO (with a mandate to protect and promote freedom of expression) and UNICEF (in its focus on vaccination and immunisation). While different types of harmful information, including misinformation, disinformation and hate speech were countered by these agencies, there was a lack of effective collaboration among them and their external partners. A review of the UN system indicates the lack of a common understanding as a key limitation (Delsol and Trithart 2023). Other challenges encountered include inadequate capacity to analyse and manage the infodemic, inability to conduct impact assessments in different contexts and difficulty to work effectively with large technology platforms.

As a normative concern, the focus on equity in the PA should also mean that the requirements of epistemic justice are observed in all measures and decisions pertaining to pandemic prevention, preparedness and response, including infodemic management. Since informational needs tend to escalate during a serious public health event, greater attention needs to be paid to epistemic harms and injustices that could arise, especially for the already disenfranchised and/or marginalised. Yet, not enough is said about how such measures or decisions—and the social epistemology that underscores them—could properly be accounted for, and how the needs and interests of individuals, different groups and the ethical values of knowledge-producing institutions be weighed up.

Our proposal is that the PA should set out an epistemic architecture, which we broadly define as a non-exclusive blueprint or layout that identifies epistemic infrastructures within which epistemic attitudes are formed, and how these infrastructures are (or should be) interlinked (Nijsingh and van Bergen 2019, 738). It follows that a key goal of this epistemic architecture is to provide guidance on which epistemic infrastructures are relevant to infodemics and how they could be applied together. An epistemic infrastructure may be understood as the “devices, methods, equipment, concepts and rules for knowledge production” of a knowledge organisation (Gustafsson and Lidskog 2023, 4), an epistemic community (Shin et al. 2025; Berg and Lidskog 2024), or society (Yoo 2024). Fact-checking, as Shin et al. (2025, 3) explain, may be conceptualised as an epistemic infrastructure as it “represents an active and contested space of intervention, where foundational questions about truth, authority, and credibility are negotiated through shifting combinations of human judgment, institutional norms, and technological mediation”. In this view, an epistemic infrastructure relates to the sociotechnical systems, institutional arrangements and procedural norms that shape knowledge production, evaluation and circulation through fact-checking. A similarly broad definition is applied by Youngjin Yoo (2024) in his evaluation of how scientific journals, as a crucial component of an epistemic infrastructure, are impacted by generative AI. In his evaluation, an epistemic infrastructure encompasses “the foundational systems, institutions and mechanisms that underpin the creation, validation, dissemination, and consumption of knowledge within our society” (Yoo 2024, 137). Our definition of epistemic infrastructure is narrower in that it focuses on the WHO as a knowledge organisation (i.e. similar to that applied by Gustafsson and Lidskog 2023; Berg and Lidskog 2024), and on the normative aspects of knowledge production.

As a knowledge organisation, the epistemic infrastructure of the WHO on infodemics as a technical concern is vast and complex. At first blush, the normative aspects are much more rudimentary in comparison since there is just one published ethical guidance on social listening in infodemic management (WHO 2025b). Delving deeper into the substance of this ethical guidance, however, it will quickly become clear that the ethical framework that it sets out should be read and applied alongside other normative frameworks that are issued, not only by the WHO but also by other UN agencies and organisations. For instance, the human rights framework on public health emergencies of the Global Health Law Consortium and the International Commission of Jurists (GHLC-ICJ Framework) (Habibi et al. 2023) is one such normative framework that has been referenced. While this human rights framework does not make explicit reference to infodemics, its discussion on the application of digital technologies in response to a public health emergency clearly recognises that harms and challenges are linked to this phenomenon (in Article 18, for instance). A critical contribution of the GHLC-ICJ Framework is that it could forge a common and more holistic understanding of infodemic-related harm. In Section V, for instance, the rights-based approach is not confined to a single human right obligation but takes into account limitations and derogations to human rights (e.g. the right to freedom of expression, the right to freedom of association and the right to peaceful assembly). Just as important is its emphasis on having strict regard for the principles of legality, necessity, proportionality and non-discrimination. Given that all UN agencies have mandates that give differential emphasis to one or more human right obligations, the GHLC-ICJ Framework could help bridge epistemic and practical differences.

It also seems to us that the GHLC-ICJ Framework is epistemically more open than the PA. Whereas the latter limits public health decisions and related measures to “the best available science and evidence” (Article 3(6)), the former highlights that States must “consult and take into account the self-expressed needs, knowledge, expertise and perspectives of rights holders”, and “guarantee effective and institutionalized public participation and deliberation mechanisms which are accessible to everyone, in order to meet its human rights obligation of respecting and ensuring meaningful and effective participation” (GHLC-ICJ Framework, Article 7). Additionally, epistemic accountability is more pronounced in the GHLC-ICJ Framework in its specification of the human rights duties relating to non-State actors, such as social media companies where infodemics is concerned (GHLC-ICJ Framework, Article 5). Non-state actors, particularly business enterprises, have a duty to respect and, where applicable, to contribute to the fulfilment of human rights. They should, where relevant, “proactively engage, collaborate and coordinate with States, individually and collectively, to ensure the full realization of health and human rights” (GHLC-ICJ Framework, Article 5(b)(ii)). This statement is especially pertinent in the light of the challenges that UN agencies faced in the lack of resources to pay full-price to social media platforms for advertising UN messages about COVID-19, and to customise social listening tools (initially developed for marketing) for public health purposes. While there are obvious synergies between substantive normative and procedural principles set out in the ethics guidance of WHO on social listening and the GHLC-ICJ Framework, it is still unclear how these two evaluative approaches, even if centred around epistemic justice, could be interconnected.

It is beyond the scope of this paper to map out all the normative frameworks that underscore the epistemic infrastructure on infodemics of the WHO and other allied organisations. Our argument is that if the PA seeks to address the challenge of infodemics, as its preamble indicates, and that any approach adopted should advance equity, then the requirements of epistemic justice should also be addressed. This could be done by setting out an epistemic architecture in the PA (as an annex, for instance), or in an additional protocol to the PA. A key goal of this epistemic architecture is to provide High Contracting Parties with guidance as to what epistemic infrastructures are applicable and how they may be brought together to meet the challenge of infodemics, and in a manner that is consistent with epistemic justice. A practical benefit of this is that High Contracting Parties will be better able to draw on epistemic infrastructures across the different UN agencies. Where the normative aspects are concerned, we have illustrated how an epistemic architecture could interconnect different normative frameworks, such as the WHO’s ethical framework on social listening with the GHLC-ICJ Framework. Presently, the PA does not indicate how its provisions relate to international human rights law, other than an exhortation for High Contracting Parties to fully respect the dignity, human rights and fundamental freedoms of all persons (PA, Article 3(2)). Arguably, ethical commitments like equity and solidarity are treated no differently (PA, Articles 2(1), 3(4) and 3(5)). Unless there are concrete means of meeting these commitments, the value of these provisions is effectively ceremonial.

## Epistemic Complexity of Antimicrobial Resistance

In the penultimate section, we discuss the extent to which the PA applies to serious health threats like AMR. Like infodemics, AMR is identified as a challenge that it seeks to address, but the provisions do not clearly indicate how this is to be done. Our argument here is that clearly setting out an epistemic architecture could help to address an epistemically complex phenomenon like AMR. As we have noted above, an important development in the PA is its focus beyond human health in adopting a One Health approach. Whereas surveillance in the context of the IHR is narrowly focused on human health, the PA seeks to strengthen measures and capacities for multisectoral surveillance that encompasses animal and environmental health. Such surveillance measures and capacities have been set out as including:

1. For the purposes of preventing the emergence or re-emergence of infectious diseases, identifying and responding to drivers of infectious disease at the human-animal-environment interface (Article 4(a))
2. Identifying and reducing pandemic risks associated with human-animal interactions, while recognising the importance of safeguarding communities’ livelihoods and food security (Article 4(b))
3. Coordinating multisectoral surveillance to detect and conduct risk assessment of pathogens with pandemic potential, including those pathogens that may present significant risks of zoonotic spillover and those pathogens that are resistant to *antimicrobial* agents, as well as sharing of the outputs of relevant surveillance and risk assessments among relevant sectors within its territory to enhance early detection (Article 4(c), emphasis added)
4. Early detection and control measures at the community level, through strengthening mechanisms and enhancing capacities at the community level, to prevent, detect and report unusual public health events to relevant authorities within its territory, in order to facilitate actions for early containment at the source (Article 4(d))
5. Addressing public health risks associated with the emergence and spread of pathogens that are resistant to *antimicrobial* agents, facilitating affordable and equitable access to *antimicrobials* and promoting appropriate, prudent and responsible use across relevant sectors (Article 4(j), emphasis added)

This expansive approach to surveillance is emphasised in expressed recognition of “a range of environmental, climatic, social, anthropogenic and economic factors, including hunger and poverty, [which] may increase the risk of pandemics, and [High Contracting Parties] shall endeavour to consider these factors in the development and implementation of relevant policies, strategies, plans, and/or measures, at the international, regional and national levels as appropriate, in accordance with national and/or domestic law and applicable international law” (Article 4(3)).

Despite the references to One Health and to antimicrobials, AMR is not strictly speaking a “disease outbreak”, or, arguably, a public health emergency. Certain provisions, like sustained investment and support for research institutions and networks (in Article 9(2)(a)), appear to be limited to a pandemic emergency, although public health priorities are also mentioned. A “pandemic emergency” is in turn defined as “a public health emergency of international concern, that is caused by a communicable disease and: (i) has, or is at high risk of having, wide geographical spread to and within multiple States; and (ii) is exceeding, or is at high risk of exceeding, the capacity of health systems to respond in those States; and (iii) is causing, or is at high risk of causing, substantial social and/or economic disruption, including disruption to international traffic and trade; and (iv) requires rapid, equitable and enhanced coordinated international action, with whole-of-government and whole-of-society approaches” (PA, Article 1(c)). The term pandemic has not been legally defined although characteristics to which it relates have been identified as the global geographical spread of an infectious disease, in respect to which most humans have little or no existing immunity. This may be contrasted with the declaration of a public health emergency of international concern (PHEIC), defined in Article 1 of the IHR as an extraordinary event determined to constitute a public health risk to other States through the international spread of disease and to potentially require a coordinated international response. As Villarreal (2021) explains, pandemic serves a descriptive purpose in communicating the presence of a communicable disease in different parts of the world, and is thereby wider in scope as an assessment of ongoing facts. In contrast, PHEIC reflects risk-based analysis and is not linked to the presence of a disease in multiple places around the world. It also appears to operate on a binary of a public health emergency or non-emergency. In this sense, all pandemics would be PHEICs, but not all PHEICs are pandemics (Villarreal 2021, 615).

In the context of an emergency, significant risk and epistemic/knowledge gaps provide justifications for using surveillance and eHealth data for public health purposes. However, the distinction between routine data collection and that of a public health emergency is increasingly marginal as Big Data approaches draw on large amounts of data from varied data repositories (Kilgallon et al. 2022; Hutchinson and Johnson 2023; Lipworth et al. 2017). AMR surveillance is an example where modern surveillance technologies are used to collect and analyse health and non-health (e.g. environmental) data in order to inform, develop and/or implement public health interventions (see for instance, Chen et al. 2025). While AMR is clearly a serious health threat that is expected to claim many lives in the foreseeable future (Naghavi et al. 2024), there are complex epistemic challenges that limit individual and collective responses to harmful information and related concerns about AMR. Niels Nijsingh and van Bergen (2019) note that there are at least three reasons for the complexity. First, the different factors that contribute to AMR may not be apparent or linked together in an obvious manner. Second, it is a challenge that escalates over time, rather than one that exacts a heavy toll within a short and defined time period. It is, in this sense, difficult to observe or prioritise. Third, AMR does not fall neatly within a single knowledge domain, and the attitude of an actor (e.g. a healthcare provider) towards a foreign knowledge system (e.g. trade-related aspects of antimicrobials) is highly variable. Given the complexity, it should perhaps not be surprising to find that communication about AMR in traditional media has been linked to a greater likelihood of both AMR misinformation and misuse (Groshek et al. 2018). Greater online social media consumption, social media activity and reliance on social networking sites following the outbreak of COVID-19 is likely to have exacerbated the problem. Findings from another study, conducted in a manner that is akin to social listening, suggest that Twitter users between December 2018 and September 2021 showed a stronger focus on specific pathogens that do not correspond with the pathogens’ status on the WHO priority pathogen list (Kim et al. 2023). Healthcare providers who rely on Twitter or similar social networking sites for readily accessible and fast information could be misinformed as to which antimicrobial-resistant pathogens are of especial concern.

The need for more targeted public health strategies to raise awareness about AMR among specific pathogens is a well-recognised one. The PA requires High Contracting Parties to “take measures to strengthen science, public health and pandemic literacy in the population, as well as access to transparent, timely, accurate, science-and evidence-based information on pandemics and their causes, impacts and drivers, as well as on the efficacy and safety of pandemic-related health products, particularly through risk communication and effective community-level engagement” (Article 16(1)). It also requires High Contracting Parties to “conduct research and inform policies on factors that hinder or strengthen adherence to public health and social measures in a pandemic and trust in science and public health institutions, authorities and agencies” (Article 16(2)). In meeting these obligations, the WHO is to, on request, provide technical support (Article 16(3)) and facilitate collaboration with relevant organisations (Article 17). These obligations are not new, and these provisions mainly serve to formalise commitments set out in the Global Action Plan on AMR (WHO 2016; Ho and Lee 2020). If we are correct, these measures are unlikely to change the status quo.

To be effective, the PA must concretely acknowledge AMR and infodemics (whether linked to AMR or not) as a collective action problem and set out clear implementation mechanisms to address them (Hoffman et al. 2019; Kahn and Dunham 2023). The GHLC-ICJ Framework reminds us that we must move beyond awareness-raising to collaborative action. In order to build a shared sense of motivation and urgency, a just and equitable epistemic environment is needed to support active involvement of the public in a wide spectrum of engagement activities and to build trust through various methods, including online social listening strategies and working with local communities to adapt messaging (Scott-Dearing et al. 2025). In essence, if the PA is to implement a just transition in global response to challenges like AMR and infodemics, it must put in place a blueprint of an epistemic architecture, as we have proposed above. There are different ways by which detailed provisions to this effect could be incorporated into, or otherwise as part of, the PA. Any such approach should be consistent with the requirements of epistemic justice, notably in avoiding epistemic oppression and other epistemic harms, cultivating epistemic goods and ensuring equitable access to knowledge and participation in knowledge production, especially of the historically marginalised. It is beyond the scope of this paper to set out what the content of this epistemic architecture should be, although our discussion of the European Union’s AI Act may be illustrative of what it could include (Ho and Caals 2024). Additionally, other initiatives like the Just Transitions for AMR Working Group (2024) provide a cross-disciplinary perspective as to what an equitable and sustainable future with antimicrobials could look like.

## Conclusion

We are concerned that the recently adopted WHO Pandemic Agreement applies equity narrowly as distributive justice, neglecting epistemic justice. We therefore argue that the PA framework needs epistemic architectures, and use infodemics and antimicrobial resistance (AMR) as examples. With the mainstreaming of digital technology across many spheres of social life, infodemic management must be an integral part of public health emergency prevention, preparedness, response and recovery in order to avoid the abrupt escalation of health inequalities, particularly among the marginalised and disadvantaged, as witnessed during the COVID-19 pandemic. The epistemic architecture of infodemics will need to be set out clearly in the PA framework, either in a manner that is similar to the PABS (i.e. by setting out explicit terms in an annex to the PA) or through the adoption of an additional protocol. In particular, the PA could help to draw together different normative and human rights approaches and frameworks to meet the requirements of epistemic justice. A similar challenge applies to AMR as an epistemically complex phenomenon, and we argue that a global response to AMR will require a just and equitable epistemic architecture that the PA could lay the foundation for.

## Footnotes

1 We do not proffer a strong view as to whether the PA should present one epistemic architecture or if it should encapsulate multiple epistemic architectures. As a practical matter, we consider the latter to be more consistent with non-ideal epistemology (McKenna 2023).

2 The draft instrument that was considered by the INB in 2023 sets out a similar definition but goes further to highlight, as concerns, the confusion and risk-taking behaviours that can cause harm, as well as the mistrust in health authorities that infodemic causes, and its undermining of public health response (WHO 2023, Article 1(b)).
